# Impaired Attentional Disengagement from Stimuli Matching the Contents of Working Memory in Social Anxiety

**DOI:** 10.1371/journal.pone.0047221

**Published:** 2012-10-12

**Authors:** Jun Moriya, Yoshinori Sugiura

**Affiliations:** 1 School of Integrated Arts and Sciences, Hiroshima University, Hiroshima, Japan; 2 Department of Experimental Clinical and Health Psychology, Ghent University, Ghent, Belgium; Goldsmiths, University of London, United Kingdom

## Abstract

Although many cognitive models in anxiety propose that an impaired top-down control enhances the processing of task-irrelevant stimuli, few studies have paid attention to task-irrelevant stimuli under a cognitive load task. In the present study, we investigated the effects of the working memory load on attention to task-irrelevant stimuli in trait social anxiety. The results showed that as trait social anxiety increased, participants were unable to disengage from task-irrelevant stimuli identical to the memory cue under low and high working memory loads. Impaired attentional disengagement was positively correlated with trait social anxiety. This impaired attentional disengagement was related to trait social anxiety, but not state anxiety. Our findings suggest that socially anxious people have difficulty in disengaging attention from a task-irrelevant memory cue owing to an impaired top-down control under a working memory load.

## Introduction

A tendency to selectively attend to negative information is a risk factor for the development of anxiety [1]–[3]. Selective attention to threatening stimuli increases state anxiety during stressful events [4]–[7]. Cognitive models in anxiety propose that the attentional bias is derived from impaired top-down control and enhanced bottom-up attention [8]–[11]. Top-down control supports the processing of task-relevant stimuli, whereas bottom-up attention enhances the processing of task-irrelevant salient stimuli [12], [13]. Since anxious people cannot enhance the processing of task-relevant stimuli owing to an impaired top-down control, they might direct attention to task-irrelevant threatening stimuli in a bottom-up fashion. Many previous studies have revealed the impaired top-down attentional control and enhanced bottom-up attention for threatening stimuli in anxiety through both behavioral and neuroimaging tasks [14]–[17].

The cognitive models of attentional bias could apply to not only emotional but also non-emotional stimuli. That is, anxious people direct attention to non-threatening, but salient, task-irrelevant stimuli (e.g., a high-contrast flash) because of impaired top-down and enhanced bottom-up attention [18]–[26]. For example, when participants were required to process the task-relevant stimulus among task-irrelevant distractors (e.g., a flanker task), anxious and socially anxious people were strongly influenced by emotionally neutral task-irrelevant distractors [20], [21], [23], [24], [26], and they demonstrated an impoverished employment of prefrontal attentional control mechanisms [18]. An impaired top-down control might make individuals with anxiety direct attention to more salient task-irrelevant stimuli.

The processing of task-irrelevant distractors depends on the level of the load (e.g., working memory load) on top-down control [27]–[30]. Because the working memory load disrupts top-down attention, which controls interference by task-irrelevant distractors, visual attention to task-irrelevant stimuli is observed under a working memory load task. The impaired top-down control in individuals with anxiety might be influenced by working memory load. However, few studies have investigated the effects of the working memory load on visual attention to task-irrelevant distractors in anxiety [31]. Considering the interaction between top-down control mechanisms and visual attention, it is necessary to clearly indicate how impaired top-down control can change visual attention in anxiety and to clarify whether this impairment attracts attention to non-emotional task-irrelevant stimuli under a working memory load.

**Figure 1:**
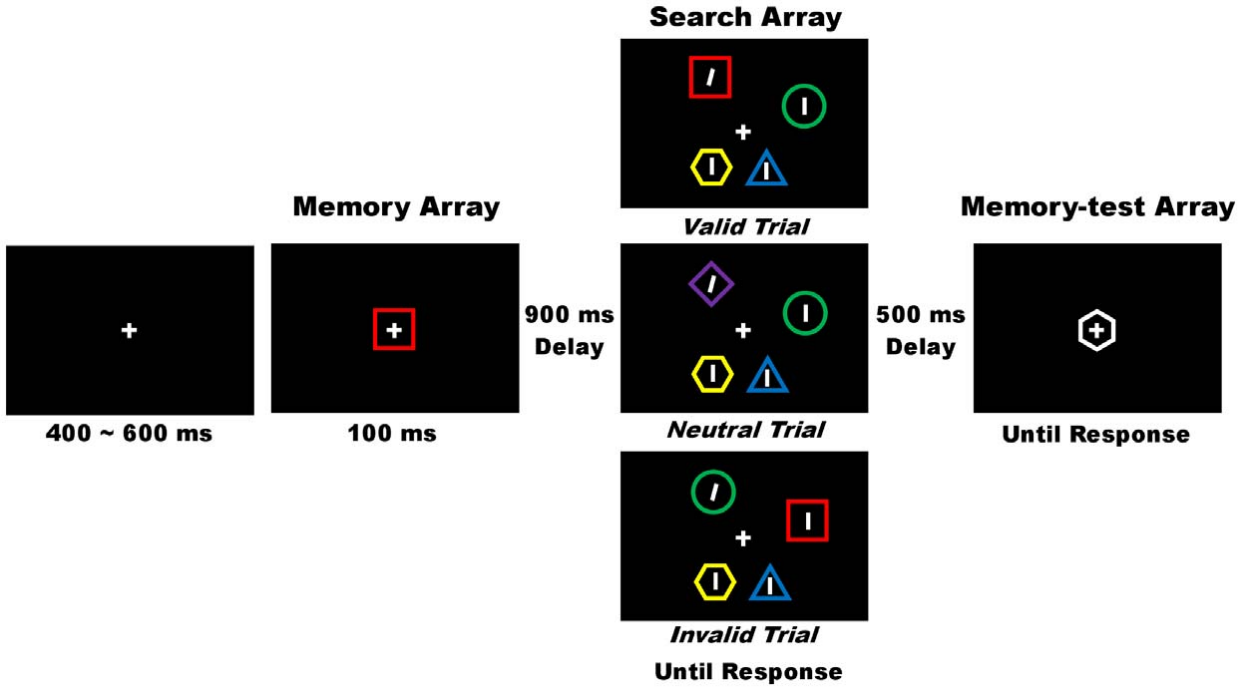
Sequence of the task under low working memory load.

Previous studies have shown the effects of working memory load on visual attention. When people hold information in their working memory, they will direct attention to the task-irrelevant stimuli matching the contents of their working memory [32]–[38]. In a study by Soto et al. [36], participants were instructed to hold a colored object as a working memory task before performing a visual search test (Figure 1). For the visual search task, participants were required to discriminate a tilted line among several vertical lines, all of which were presented among various colored objects. Three conditions were created: the tilted line was presented in a stimulus matching the contents of the working memory (valid condition), the tilted line was not presented in a stimulus matching the contents of the working memory (invalid condition), and a stimulus matching the contents of the working memory was not presented (neutral condition). The authors showed that the stimuli held in the working memory captured attention and that reaction times (RTs) for the tilted target in the valid condition were shorter than those in the invalid condition, that is, a cue validity effect. Working memory enhances task-relevant processing and enables people to focus on the target efficiently. However, people with impaired prefrontal top-down control have difficulty in compartmentalizing their working memory, that is, keeping irrelevant information separate from searching a template [37], [39]. Therefore, they direct attention to the stimuli matching the contents in their working memory even if they are task-irrelevant, and the cue validity effect is enhanced [32], [33], [35], [36], [38]. This task can investigate the effects of working memory load on visual attention to task-irrelevant stimuli.

For the present research, we examined the effects of working memory load on visual attention to investigate whether impaired top-down control will make socially anxious people direct attention to task-irrelevant stimuli by using a paradigm put forth by Soto et al. [36]. We focused on trait social anxiety because impaired attentional control is strongly associated with trait social anxiety [40]. Considering that people with an impaired top-down control show a strong effect of working memory load on visual attention to task-irrelevant stimuli [39], we hypothesized that greater attention to task-irrelevant stimuli would be observed as trait social anxiety increases. Attention to task-irrelevant stimuli was divided into two operations: engagement and disengagement. Recent studies have shown that individuals with trait anxiety have difficulty particularly in disengaging their attention from both emotional [41]–[49] and non-emotional stimuli [24]. Therefore, we hypothesized that impaired attentional disengagement from task-irrelevant stimuli would be observed in individuals with trait social anxiety.

According to the attentional control theory of Eysenck et al. [11], enhanced processing of task-irrelevant stimuli in anxiety was observed under an increased load because top-down control was more impaired. When working memory load increases, delayed disengagement might appear prominently in individuals with high trait social anxiety. Recently, Bishop [18] showed that high trait-anxious individuals showed decreased efficiency of top-down control under only low load but not high load [50], and she insisted that these results were inconsistent with the attentional control theory. However, she manipulated perceptual load, not working memory load. According to the load theory [28], [51], the effects of working memory load and perceptual load on top-down control are different. While increasing working memory load disrupts top-down control, increasing perceptual load does not do so, because the perceptual load affects limited perceptual processing capacity rather than cognitive control [29], [30]. Therefore, high perceptual load in Bishop’s study [18], [50] did not affect attention to task-irrelevant distractors in high trait anxiety. It is still unclear whether increasing working memory load enhances the processing of non-emotional task-irrelevant stimuli in high trait social anxiety. We manipulated working memory load by asking participants to memorize one object or two objects. Top-down control would be disrupted with the increased load task. As a result, impaired attentional disengagement (i.e., delayed disengagement from the memory cue) might be positively correlated with trait social anxiety especially under high working memory load.

Manipulating working memory load is also important for investigating the effects of visual working memory capacity in trait social anxiety. Attention to task-irrelevant stimuli is diminished under high working memory load because of limited working memory capacity [38], [52], [53]. High working memory load leads to increased competition between working memory stimuli and attenuates memory representations [12]. A recent study also showed that the attentional network does not work if the prefrontal cortex is preoccupied in maintaining several stimuli in memory [52]. The results indicated that memory-matching stimuli did not capture attention under high working memory load. If working memory capacity is diminished in high trait social anxiety, memory representations might be impoverished by competition in working memory under high working memory load. In the results, attention to task-irrelevant stimuli is diminished under high working memory load. Although reduced working memory capacity is considered to be associated with trait anxiety [54], [55], a recent study showed that individuals experiencing high trait levels of social anxiety have high visual working memory capacity [56]. Therefore, we hypothesized that even under high working memory load, attention to task-irrelevant stimuli might be observed in individuals with high trait social anxiety.

We also focused on the differences among trait social anxiety, state anxiety, and other negative emotional states (e.g., depression). Trait anxiety is considered a personality position, whereas state anxiety refers to the current level of anxiety. Recent studies have shown that impaired top-down attention is associated particularly with trait anxiety while state anxiety is associated with increased bottom-up attention to salient stimuli [8], [9], [23]. Considering that the working memory load affects visual attention to task-irrelevant stimuli in individuals with impaired top-down control, trait social anxiety might predict delayed attentional disengagement. On the other hand, state anxiety and depression might not predict attentional disengagement.

## Methods

### Participants

The participants were 46 undergraduate students (11 males and 35 females) who provided informed consent. All reported normal or corrected-to-normal vision.

### Questionnaires

#### Brief Fear of Negative Evaluation Scale (BFNE; [57], [58])

The BFNE Scale assesses apprehension related to others’ negative evaluations and reflects the degree of trait social anxiety. The scale comprises 12 items and uses a 5-point Likert scale. The scale has high internal consistency (Cronbach’s alpha = .92), and a test–retest reliability with a three-month interval (*r* = .74).

#### Social Phobia Scale (SPS; [59], [60])

The SPS assesses the fear of scrutiny from other people, which is also a feature of trait social anxiety. The scale comprises 20 items and uses a 5-point Likert scale. The scale has high internal consistency (Cronbach’s alpha = .91), and a test–retest reliability with a five-month interval (*r* = .72).

#### State-Trait Anxiety Inventory-State Form (STAI-S; [61], [62])

The STAI-S was used to measure the degree of state anxiety. The scale comprises 20 items and uses a 4-point Likert scale. The scale has high internal consistency (Cronbach’s alpha = .87), and a test–retest reliability with a three-month interval (*r* = .80).

#### Self-Rating Depression Scale (SDS; [63], [64])

The SDS was used to measure the degree of depressive symptoms. The scale comprises 20 items and uses a 4-point Likert scale. It has high internal consistency (Spearman-Brown split-half coefficient = .73) and a test–retest reliability with a one-week interval (*r* = .85).

### Stimuli and Apparatus

All stimuli were presented against a black background. In the memory and memory-test arrays, participants were required to focus on a fixation cross (with a visual angle of 0.17°×0.17°) that appeared at the center of a screen. The memory cue could be any of the following: a circle, diamond, square, triangle, hexagon, or second hexagon with a 90° rotation. All visual angles were 1.8°×1.8°. The color of the cue was red, green, blue, yellow, violet, or white.

In the visual search array, four colored objects appeared on an imaginary circle at a fixation with a radius of 4.0°. Each object could be positioned at one of eight possible locations on this imaginary circle. Each stimulus was unique in color and shape, and one line was located inside each colored object. Each line was 0.52° long and 0.12° wide. Among the lines, three were vertically placed and the fourth (i.e., the search target) was randomly tilted at 15° either to the left or to the right.

The stimuli were presented on a 17-inch monitor. The experiment was programmed using MATLAB equipped with Psychophysics Toolbox [65], [66]. The viewing distance was about 60 cm.

### Procedure

Figure 1 presents an example of an experimental trial. The participants were seated in front of the monitor in a dim room. A fixation cross appeared at the center of the screen for 400–600 ms, and participants were required to focus on the cross. Following fixation, the memory cue was presented at the center of the screen for 100 ms in the memory array. Under low working memory load, only one memory cue was presented whereas under high working memory load, two memory cues were located 1.8° to the left and right of the fixation. Participants were instructed to memorize both the color and shape of the cues and to keep them in mind throughout the entire trial. After a delay of 900 ms, the search stimuli appeared on an imaginary circle in the visual search array. Participants were instructed to discriminate the orientation of the target and to press the appropriate key, using the left hand for left-oriented lines and the right hand for right-oriented lines. After their response, followed by a 500-ms interval, there was a memory-test array. Under low working memory load, one colored object was presented whereas under high working memory load, two colored objects were presented. Participants were instructed to indicate whether both the color and shape of the colored objects were identical to those of the memory cues by pressing one of the two appropriate keys. Under high working memory load, the two objects were identical to the memory cues on trials involving the same objects. However, on trials involving different objects, one of the objects could differ in color, shape, or both from the memory cue, whereas the other stimulus remained the same. The intertrial interval was 500 ms.

After 24 practice trials for each load, each participant completed 2 blocks (high and low working memory loads) and 108 trials per block. The order of the blocks was randomized for each participant. There were three different types of trials in the visual search array, each defined by the validity of the memory cue. On the valid trials, the tilted target appeared within the colored object identical to the memory cue under low working memory load, and either one of the memory cues reappeared with the tilted target line within it under high working memory load. On the invalid trials, the memory cue under low working memory load or either one of the memory cues under high working memory load was re-presented in the search display but always contained a non-target line rather than the actual target. On the neutral trials, the memory cues were not present in the visual search array, and the features of the stimuli presented in the array did not match those of the cue. Trial type was randomly determined, and each trial type had an equal probability of occurrence. The participants were informed that only accuracy would be examined in the memory task and they were instructed to respond as accurately and as quickly as possible in the visual search task. At the end of the task, the participants were required to complete all the aforementioned scales.

### Analysis

Previous studies have shown that attentional engagement is associated with short RTs in a valid condition compared with RTs in a neutral condition, whereas attentional disengagement is associated with long RTs in an invalid condition compared with RTs in a neutral condition [67], [68]. On the basis of these studies, we calculated attentional disengagement from memorized stimuli (i.e., RTs in invalid conditions – RTs in neutral conditions) and attentional engagement to memorized stimuli (i.e., RTs in neutral conditions – RTs in valid conditions).

## Results

Visual search and working memory accuracies are shown in Table 1. They were analyzed using a two-way ANOVA of validity (valid, neutral, invalid) and working memory load (high, low). Main effects and interaction were not observed in the visual search accuracy (main effect of validity: *F*(2, 90) = 2.74, *p* = .07, *η^2^_p_* = .06; main effect of load: *F*(1, 45) = 0.20, *p*>.10, *η^2^_p_* = .01; interaction: *F*(2, 90) = 1.21, *p*>.10, *η^2^_p_* = .03). In terms of working memory accuracy, the ANOVA revealed the main effects of validity (*F*(2, 90) = 5.20, *p*<.01, *η^2^_p_* = .10), and working memory load (*F*(1, 45) = 119.00, *p*<.001, *η^2^_p_* = .73). The interaction was not significant. The accuracy rates for the valid trials were higher than those for the neutral and invalid trials (*p*<.01). In addition, the accuracy rates under low working memory load were higher than those under high working memory load.

**Table 1:** Mean Percentages of Accuracy Rates (Standard Deviations in Parentheses) in Visual Search and Working Memory Tasks.

	Visual Search Task			Working Memory Task		
	Valid	Neutral	Invalid	Valid	Neutral	Invalid
Low Working Memory Load	99.6 (1.4)	99.2 (1.7)	98.8 (2.0)	95.6 (3.9)	94.0 (5.4)	95.2 (5.0)
High Working Memory Load	99.4 (1.7)	99.3 (1.9)	99.2 (1.8)	89.5 (6.2)	86.5 (6.9)	86.2 (7.2)

**Figure 2:**
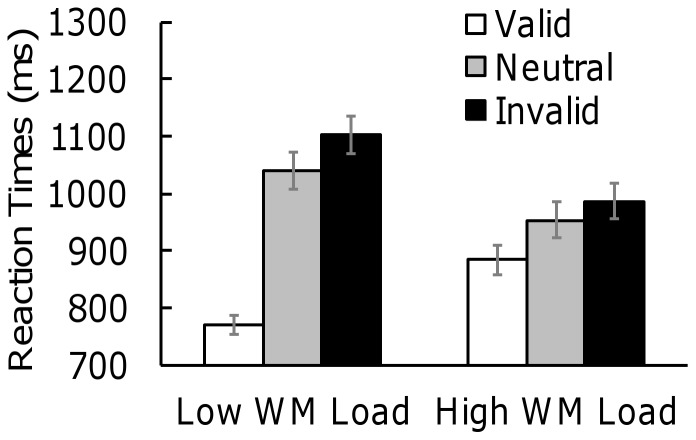
Mean correct RTs (ms) in a visual search task. Note. Error bars represent standard errors. WM = Working Memory.

We analyzed RTs in the visual search array (Figure 2). Incorrect responses and trials where RTs were more than three standard deviations from the mean were excluded. The average proportion of outliers was 1.4%. We conducted a two-way ANOVA of validity and working memory load. The main effect of validity was significant (*F*(2, 90) = 152.79, *p*<.001, *η^2^_p_* = .77). There was also a two-way interaction effect (*F*(2, 90) = 83.16, *p*<.001, *η^2^_p_* = .65). The main effect of working memory load was not significant. A Bonferroni-corrected simple effects test (two-tailed) revealed that, under both high and low working memory loads, the RTs for the valid trials were shorter than those for the neutral and invalid trials (neutral: *p*<.001; invalid: *p*<.001) and that the RTs for the neutral trials were shorter than those for the invalid trials (*p*<.001). The important finding is that in the valid trials, the RTs under high working memory load were longer than those under low working memory load (*p*<.001), while in the neutral and invalid trials, the RTs under high working memory load were shorter than those under low working memory load (neutral: *p*<.001; invalid: *p*<.001). If participants directed attention to the task-irrelevant stimuli matching the contents of their working memory, RTs in the valid condition became short and RTs in the invalid condition became long. These results showed that increasing working memory load decreased the effects of attention to task-irrelevant stimuli. Under high working memory load, participants did not direct attention to task-irrelevant memory-matching stimuli compared to under low working memory load.

We investigated the relationships between trait social anxiety and the effects of engagement with the memory cue (RTs neutral – RTs valid), and disengagement from the memory cue (RTs invalid – RTs neutral) in the visual search task. Table 2 shows the correlations between the indices and each scale, as well as correlations among scales. Positive scores of engagement and disengagement indicate enhanced engagement to the memory cue, and impaired disengagement from the memory cue respectively. Only trait social anxiety (i.e., BFNE and SPS scores) was positively correlated with the attentional disengagement index under both low and high working memory loads.

**Table 2:** Correlations between Attentional Indices and Scales.

	Disengagement in low load	Engagement in high load	Disengagement in high load	BFNE	SPS	STAI-S	SDS
Engagement in low load	–.16	.28	.07	–.19	–.02	–.04	–.01
Disengagement in low load	–	–.01	.24	.39**	.30*	.19	.24
Engagement in high load		–	–.49**	–.18	–.07	–.22	–.25
Disengagement in high load			–	.41**	.38**	.06	.29
BFNE				–	.60***	.11	.33*
SPS					–	.38**	.61***
STAI-S						–	.60***
SDS							–

Note. BFNE = Brief Fear of Negative Evaluation Scale; SPS  =  Social Phobia Scale; STAI – S  =  State Trait Anxiety Inventory – State; SDS  =  Self-Rating Depression Scale.

* *p*<.05,

** *p*<.01,

*** *p*<.001.

We also conducted a multiple regression analysis to determine whether trait social anxiety or state anxiety contributed to attentional disengagement index in each working memory load. Because BFNE is related to the cognitive function of trait social anxiety, we used BFNE as an independent variable instead of SPS. Under low working memory load, the model was significant (*F*(2, 43) = 4.30, *p*<.05, *R^2^* = .13). Only BFNE was a statistically significant predictor of attentional disengagement (*B = *4.58, *SE B* = 1.75, *β* = .37, *p*<.05). STAI-S did not predict the index (*B = *1.52, *SE B* = 1.47, *β* = .15, *ns*). Under high working memory load, the model was also significant (*F*(2, 43) = 4.35, *p*<.05, *R^2^* = .13). BFNE was the only statistically significant predictor of attentional disengagement (*B = *3.45, *SE B* = 1.18, *β* = .41, *p*<.01). STAI-S did not predict the index (*B = *0.11, *SE B* = 0.99, *β* = .02, *ns*). There were no indications of multicollinearity, with VIF values <2 and tolerance 98.

**Figure 3:**
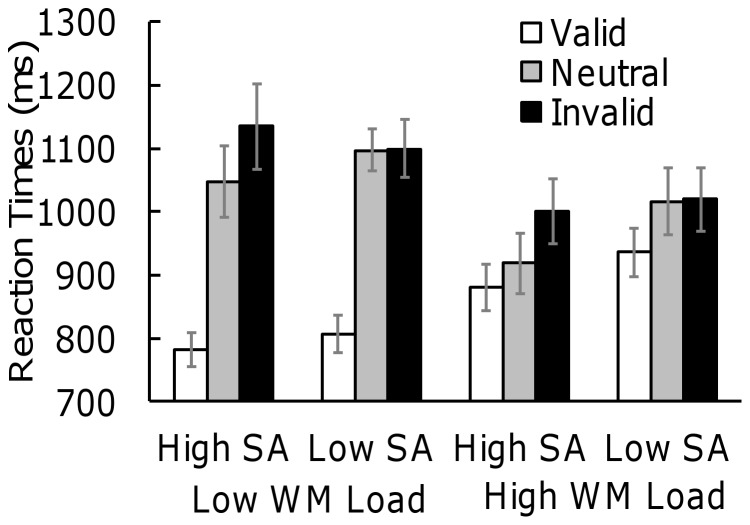
Mean correct RTs (ms) in high and low socially anxious individuals in a visual search task. Note. Error bars represent standard errors. SA = Social Anxiety; WM = Working Memory.

We also divided participants into individuals with high and low trait social anxiety in the upper and lower tertile ranges of BFNE and analyzed RTs in the visual search array (Figure 3). Seventeen participants (3 males and 14 females) had high trait social anxiety (Mean BFNE score = 50.2, *SD* = 2.6) and sixteen (4 males and 12 females) had low trait social anxiety (Mean BFNE score = 33.9, *SD* = 4.7). We conducted a three-way ANOVA of validity, working memory load, and trait social anxiety (high, low). The main effect of validity was significant (*F*(2, 62) = 101.90, *p*<.001, *η^2^_p_* = .77). We also observed significant interactions between validity and working memory load (*F*(2, 62) = 56.24, *p*<.001, *η^2^_p_* = .65), and between validity and trait social anxiety (*F*(2, 62) = 3.48, *p*<.05, *η^2^_p_* = .10). The other main effects and interactions were not significant. The interaction between validity and working memory showed the same results as mentioned above. That is, in the valid trials, the RTs under high working memory load were longer than those under low working memory load while in the neutral and invalid trials, the RTs under working memory load were shorter than those under low working memory load. The important result is the interaction between validity and trait social anxiety, which showed that in individuals with high trait social anxiety, RTs among valid, neutral, and invalid trials were significantly different (all *p*s <.001) while in individuals with low trait social anxiety, RTs between neutral and invalid trials did not significantly differ. Engagement to the memory-matching stimulus was observed in individuals with both high and low trait social anxiety. However, only those with high trait social anxiety had difficulty in disengaging attention from the stimuli, which matched the contents of working memory.

The RTs in the visual search array in each condition (i.e., valid, neutral, and invalid conditions) were not correlated with trait social anxiety, state anxiety, or depression. The accuracy rates in working memory array for each validity trial were not correlated with the scales either. The performance itself for visual search and working memory was not associated with trait social anxiety.

## Discussion

The present study investigated whether socially anxious people with impaired top-down control directed attention to task-irrelevant stimuli under a working memory load. Because people with impaired prefrontal top-down control have difficulty keeping separate irrelevant information, they will direct attention to the stimuli matching the contents in working memory even if they are task-irrelevant [37], [39]. The present study showed that as trait social anxiety increased, greater effects in visual attention by the working memory were observed. In particular, there was a delay in attentional disengagement from the task-irrelevant stimuli matched in the working memory among socially anxious people. Even when working memory load increased, impaired attentional disengagement in trait social anxiety was observed. Although many cognitive models in anxiety propose that impaired top-down attentional control enhances the processing of task-irrelevant stimuli [8]–[11], previous studies did not produce an attention to task-irrelevant stimuli under a cognitive load task in anxiety. This study elucidated that trait social anxiety had an influence on visual attention to non-emotional task-irrelevant stimuli that is identical to that of a memory cue under a working memory load.

In the present study, the impaired attentional disengagement from task-irrelevant stimuli was observed in trait social anxiety under not only low but also high working memory load. This is consistent with the attentional control theory, in which deficient top-down control is greater with a high cognitive load [10], [11], [69]. Although some previous studies have shown that trait anxiety does not have an effect on attentional control under high load [18], [50], they manipulated not working memory load but perceptual load. The effects of working memory load and perceptual load on top-down control are different [28], [51]. Contrary to the effects of perceptual load [18], [50], high working memory load did not exclude the effects of impaired attentional control in trait social anxiety, but maintained theses effects. Manipulating working memory load but not perceptual load is an appropriate way to disrupt top-down control, which inhibits interference from task-irrelevant distractors. The present results suggest that increasing working memory load disrupts top-down control especially in individuals with trait social anxiety, and that they are unable to inhibit the task-irrelevant distractors.

The impaired attentional disengagement under high working memory load in trait social anxiety also suggests that individuals with high trait social anxiety do not necessarily have a low working memory capacity. According to previous studies, the effects of task-irrelevant stimuli on visual attention are not observed under high working memory load because of limited working memory capacity [38], [52], [53]. If working memory capacity is reduced in high trait social anxiety as the previous studies showed [54], [55], the effects of task-irrelevant stimuli might not be observed. The effects of task-irrelevant stimuli under high working memory load suggest that individuals with trait social anxiety have sufficient working memory capacity. However, it is still unclear whether individuals with high trait social anxiety have more visual working memory capacity than those with low trait social anxiety as in a previous study [56], because the working memory load in the present study was not too high. Because the average visual working memory capacity is three to four simple objects [70]–[73], two stimuli in the present study might not totally deplete working memory capacity. Previous studies also showed that the working memory load of two stimuli was insufficient for the attentional guidance effect to disappear, while the effect disappeared completely with more than three stimuli in working memory load [38], [52], [53]. Future studies should increase the stimuli in working memory load to evaluate the effects of individual differences of working memory capacity in trait social anxiety.

Inconsistent with our hypothesis, impaired attentional disengagement was not enhanced in high trait social anxiety under high working memory load compared to that under low working memory load. Because high working memory load disrupts top-down control more than low working memory load, individuals with trait social anxiety might have difficulty in controlling attention under high working memory load. One possibility is that the low working memory load was enough to disrupt top-down control in individuals with trait social anxiety. Even after increasing working memory load, the effects of the load did not change. The other possibility is that the present task underestimated the effects of working memory load on visual attention in trait social anxiety. In the present task, high working memory load diminished the attention to task-irrelevant stimuli. Therefore, even if high working memory load enhances the processing of task-irrelevant stimuli in individuals with high trait social anxiety, the effects might not be observed as, for example, long RTs in invalid trials. Although the working memory load decreased attention to task-irrelevant stimuli in all participants, attentional disengagement index under high working memory load was not diminished compared to that under low working memory load in individuals with high trait social anxiety (low load: 87 ms; high load:82 ms). The invariant disengagement index might reflect the strong impaired attentional control under high working memory load. Future studies should use different visual task to reveal the effects of working memory load on attention in trait social anxiety.

We observed delayed disengagement rather than rapid engagement, from the memory cue in individuals with trait social anxiety. This is consistent with the findings of previous studies that showed that people with trait anxiety and trait social anxiety had difficulty disengaging from threatening stimuli [41]–[49]. In many previous studies that showed delayed disengagement instead of rapid engagement, a threatening stimulus was presented unilaterally [41]–[43], [49], whereas the present study presented several stimuli at the same time. Because an abrupt onset of stimuli captures attention automatically [74], these previous studies might have underestimated the effects of engagement. In fact, when presented with two stimuli at one time, individuals with high trait anxiety rapidly engage their attention to a threatening stimulus [75]. In order to reveal whether rapid engagement in trait anxiety, which was not observed in the present study, is specific to emotional stimuli, future studies should use emotional stimuli in a similar task. However, the important point in the present results is that impaired attentional disengagement in trait social anxiety was observed not only for emotional stimuli but also for non-emotional stimuli. This finding is in line with recent studies that have shown that impaired attentional control in trait anxiety was observed not only for emotional stimuli but also for non-emotional stimuli [18], [20], [21], [23], [24], [26], while few studies have shown the impaired attentional “disengagement” for non-emotional stimuli [24]. The top-down control mechanisms in trait anxiety might be generally impaired.

We also measured state anxiety and depression for participants, but these did not predict the effects of working memory load on visual attention. According to cognitive models [8], [9], trait anxiety impairs top-down control whereas state anxiety induces bottom-up attention to salient stimuli. Attention to stimuli matched in the working memory is dependent on an impaired top-down control, and enhanced bottom-up attention does not induce attentional attraction [35], [37], [39], [76], [77]. Therefore, trait social anxiety, rather than state anxiety, predicted the delayed attentional disengagement in the present study. However, we only measured trait social anxiety, and it is still unclear whether individuals with trait anxiety also show impaired attentional disengagement under working memory load. Moreover, considering that individuals with social anxiety disorders have difficulty in attentional control [41], impaired attentional disengagement might be observed among them.

There was no association between performance in the working memory task and trait social anxiety. Considering the impaired top-down control in trait social anxiety, the performance might be negatively correlated with trait social anxiety. According to Derakshan and Koster [78], people with trait anxiety who have an impaired top-down control can maintain a high performance level (i.e., response accuracy), but at the expense of a reduced processing efficiency (i.e., response latency). Our findings suggest that accuracy rates in the working memory task were not reduced by trait social anxiety.

The present study chose to manipulate the working memory load on top-down control. However, top-down control includes not only working memory but also many other functions such as executive function and effortful control. The attentional guidance from working memory also depends on several other top-down controls [35], [76], [77], [79], [80]. Since valid, neutral, and invalid trials were equally presented in the present study, it is difficult to determine whether participants would voluntarily direct attention toward the memory-matching item. Considering that all trials were presented equally, participants might not voluntarily direct attention to the stimuli that matched the content of WM. However, to accurately assess the effects of voluntary and involuntary attention, we need to manipulate the proportion of valid, neutral, and invalid trials. If there were no valid trials, participants could voluntarily inhibit the memory-matching items [79]. The goal of attending to memory-matching stimuli might also play an important role for the attentional guidance from the working memory [80]. If individuals with trait social anxiety have difficulty in voluntarily inhibiting information in working memory, they might also show impaired attentional disengagement in experiments void of valid trials. Future studies should investigate which specific top-down controls affect trait social anxiety.

In summary, we investigated the effects of impaired top-down control in trait social anxiety on visual attention to task-irrelevant stimuli under a working memory load. The ability of top-down control deteriorates under working memory load and people experience interference from task-irrelevant distractors. We showed that as trait social anxiety increased, interference from task-irrelevant stimuli also increased. Delayed attentional disengagement from the task-irrelevant stimuli matched with working memory was observed with an increase in trait social anxiety. Even when working memory load increased, impaired attentional disengagement in trait social anxiety was observed. Impaired top-down control in socially anxious people might enhance the processing of task-irrelevant stimuli under working memory load and prevent them disengaging from the stimuli.
